# Pituitary metastasis of hepatocellular carcinoma as the initial presentations: a case report and review of the literature

**DOI:** 10.3389/fonc.2023.1123855

**Published:** 2023-07-06

**Authors:** Qiang He, Feng Deng, Bowen Cai, Chao You, Songping Zheng

**Affiliations:** ^1^ Department of Neurosurgery, West China Hospital, Sichuan University, Chengdu, Sichuan, China; ^2^ Department of Hepatic Surgery, The Second Affiliated Hospital of Chengdu Medical College, Chengdu, Sichuan, China

**Keywords:** hepatocellular carcinoma, pituitary metastasizes, case report, headache, literature review

## Abstract

**Background:**

Extrahepatic metastasis of hepatocellular carcinoma (HCC) is common. However, pituitary metastasis of HCC is extremely rare. Our case may be helpful to extend the understanding of the disease.

**Case presentation:**

A 65-year-old man presented to the outpatient department for unexplained headache and ptosis for 1 month. Brain imaging showed a slight enhancement tumor in the pituitary fossa, and the endocrinological assessment showed normal results. We considered the tumor as a non-functioning pituitary adenoma before surgery. Then, the tumor was resected by an endonasal endoscopic transsphenoidal approach. The histopathological examination results revealed the pituitary metastasis of HCC. Additional abdominal imaging revealed tumors were located in the left and right liver lobes with portal vein invasion and bilateral ilium metastases. After multidisciplinary cooperation, the patient chose chemotherapy.

**Conclusion:**

We report a case of HCC metastasis to the pituitary gland that initially presented with neurological symptoms. We should consider the possibility of pituitary metastasis in HCC patients.

## Introduction

The most common primary tumors metastasizing to the pituitary gland are breast and lung cancers. The common sites of metastasis for hepatocellular carcinoma (HCC) are the lungs, lymph nodes, and rarely bones (1). Pituitary metastasis of HCC is rare and can be misdiagnosed as pituitary adenoma. In this study, we report a case of a 65-year-old man with pituitary metastasis at the initial presentation and do a comprehensive literature review to promote a better understanding of pituitary metastasis of HCC.

## Case report

A 65-year-old man with a headache and left ptosis for 1 month was admitted. He also had a left blurred vision. These symptoms gradually worsened. He declined any symptoms of abdominal pain, nausea, and vomiting. Computed tomography (CT) and magnetic resonance imaging (MRI) revealed a mass in the pituitary fossa. He denied discomforts elsewhere. He had hepatitis B antigen-positive for 6 years and did not receive antiviral treatment. His family members did not have a history of liver cirrhosis. Neurological examinations revealed left eyelid ptosis and a small left pupil. Eyeball movements in all directions were normal. The remaining physical examinations were insignificant.

The hepatitis B virus test showed a hepatitis B surface antigen level of 2813.00 COI (normal range, 0–0.9 COI). The liver enzyme test detected a glutamic pyruvic transaminase level of 46 IU/l (normal range, <40 IU/l) and a glutamyl transpeptidase level of 157 IU/l (normal range, <60 IU/l). The results of blood platelet count and coagulation function were normal. An endocrinological test revealed a normal hormone level. The results in the oncology department showed that the level of alpha-fetoprotein was 260 ng/ml (normal range, <7 ng/ml).

**Figure 1:**
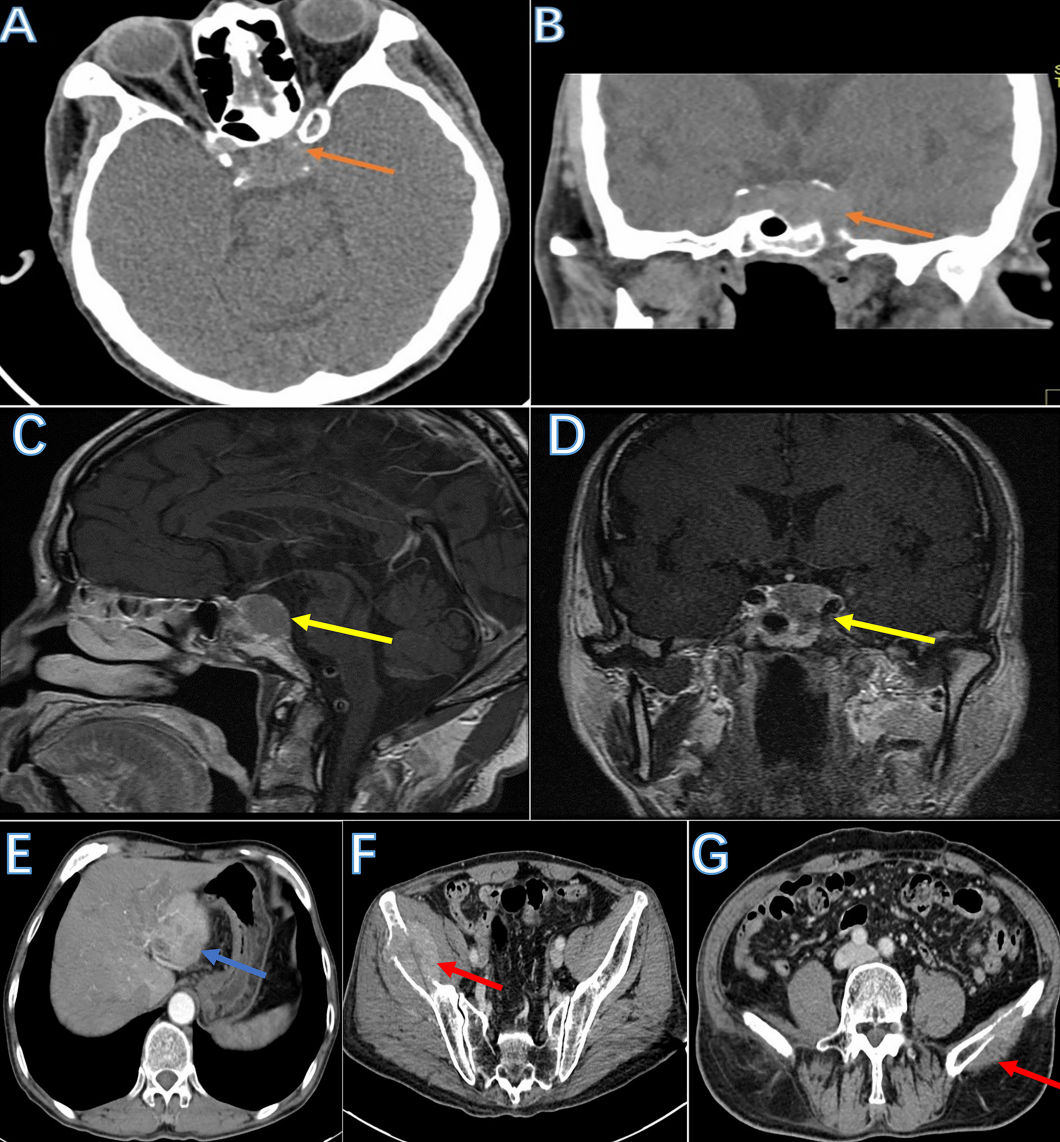
The image of the tumor. Skull base CT showed a mass with adjacent bone destruction in the sellar region (**A, B**: brown arrow). MRI showed a tumor with a clear boundary with mild enhancement after injection of a contrast agent (**C, D**: yellow arrow). The abdominal CT showed multiple tumors in the liver. Tumors are filling in the portal vein lumen (**E**: blue arrow). Bilateral iliac bones were destroyed due to metastasis (**F, G**: red arrow).

**Figure 2:**
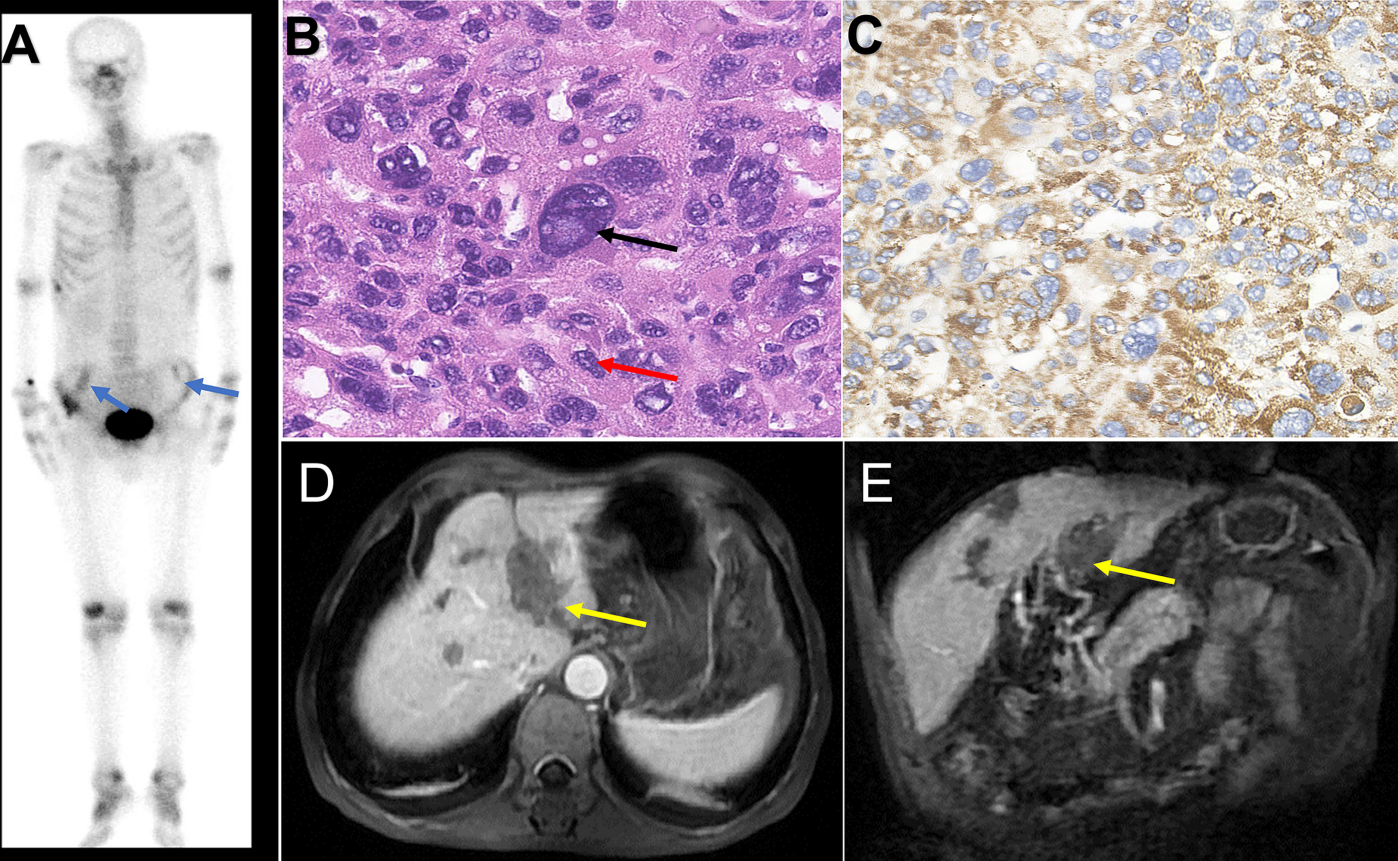
The image and pathology of lesions. The single photon emission computed tomography showed 99 mTC-MDP aggregated in the bilateral iliac bones (**A**: blue arrow). Hematoxylin and eosin staining of the pituitary tumor demonstrates a tumor with nuclei of different shapes **(B)**. Multinuclear giant cells (black arrow) and carcinoma cells (red arrow). Immunohistochemical expression of metastatic hepatic carcinoma glypican-3 **(C)**. The abdominal MRI showed multiple tumors with portal vein tumor embolus (**D, E**: yellow arrow).

CT showed a pituitary mass with adjacent bone destruction (**Figures 1A, B**). MRI revealed a tumor with enhancement in the pituitary fossa (**Figures 1C, D**). The pituitary gland was not observed in the images. The tumor led to the enlargement of the pituitary fossa and saddle bottom subsidence. No clear boundary was observed between the tumor and the surrounding tissue. The tumor also invaded the left sphenoid sinus. After removing the tumor, the abdominal CT showed multiple inhomogeneous tumors with the portal vein invasion and multiple bilateral ilium metastasis (**Figures 1E–G**), and single photon emission computed tomography (SPECT) showed high metabolism in the bilateral iliac bone (**Figure 2A**).

After completing the preoperative preparation and ruling out contraindications to surgery, we performed the tumor resection using trans-sphenoidal surgery. The tumor had a tough texture with relatively abundant blood supplement. Close adhesion between the pituitary, dura, and seller and tumor was observed. Gross total resection was performed.

The pathological results revealed that the positive markers were PCK, Hepar, and GPC-3, and the negative markers were EMA, SSTR2, PR, GH, TSH, ACTH, FSH, LH, PRL, S100, and P63 (**Figures 2B, C**). The final diagnosis was pituitary metastasis of HCC and HCC. The postoperative images showed that the tumor was completely resected (**Supplementary Figure 1**).

After the brain tumor resection, the patient’s headache subsided but still had a third cranial nerve palsy. Half a month after tumor resection, the patient was administered lenvatinib after consulting with hepatobiliary specialists. Three months after the tumor resection, he complained of pain in the liver and bilateral ilium areas. Therefore, painkillers were added. The patient was admitted due to symptoms of nausea, jaundice, and vomiting. Moreover, the patient’s weight also dropped. The abdominal MRI showed multiple tumors with obstruction of the bile duct (**Figures 2D, E**). Unfortunately, the patient passed away at home after breakfast 3.5 months after the tumor resection, and the exact cause was unknown.

## Discussion and conclusions

The pituitary metastasis of HCC is extremely rare, with less than 1% of all intracranial metastases (2). In this study, we report a case of pituitary metastasis of HCC with neurological deficits as the initial symptom. Only 11 cases of HCC metastasizing to the pituitary gland have been reported (3–12) (**Table 1**). They were mostly elderly male patients with a background of hepatitis, heavy alcohol consumption, and hepatitis B surface antigen-positive. The mean survival time was 4.94 months.

**Table 1:** Reported cases of pituitary metastasis from HCC.

Authors/year	Age/gender	Medical history	Initial manifestations	Liver function	Endocrine tests	Clinical course	Other metastasis	Treatment	Survival time (months)	Survival
Aung/2002 (9)	40/M	*HBsAg positive*	Diplopia, headache, vomiting	N	AB	Brain→ liver	NA	TS	3	Death
	71/M	*HBsAg positive*	Headache, diplopia, ptosis	N	AB	Brain→ liver	Adrenal gland	TS, radiotherapy,	12	Death
Karamouzis/2003 (10)	59/M	Heavy drinkers, gastrointestinal bleeding	Abdominal pain, fever	N	N	Liver→ brain	Multiple bone	Excision of HCC	24	Alive
Komninos/2004 (11)	68/M	Heavy smoker, alcohol abuser	Headache, anorexia, somnolence	N	AB	Brain→ liver	Pancreas, adrenal gland	TS, radiotherapy	3	Death
Hirsch/2005 (12)	46/M	NA	Sudden headache, blurred vision	N	N	Brain→ liver	Adrenal gland	TS, chemotherapy	12	Death
Perez/2007 (13)	65/M	Alcohol abuser, *HBsAg positive*	Sudden ptosis	AB	AB	Brain→ liver	NA	TS	5	Death
Takigawa/2011 (14)	58/M	Pituitary resection, hepatosplenomegaly	Diplopia, blurred vision	NA	NA	Liver→ brain	Bones	No	5	Death
Thomas/2013 (15)	50/M	Alcohol abuser	Headache, sudden diplopia	N	AB	Brain→ liver	Spine, ribs	TS	NA	NA
Tanaka/2015 (16)	80/W	Hepatitis C virus-related cirrhosis, HCC	Anorexia	AB	AB	Liver→ brain	No	No	2	Death
Rocca/2017 (17)	56/M	Alcohol abuser	Ptosis	AB	AB	Liver→ brain	No	TS	2	Death
Ambalavanan/2020 (18)	68/M	Hepatitis B, HIV, anal cancer	Headache, abdominal pain	N	AB	Liver→ brain	NA	Radiotherapy	0.5	Death

M, man; W, woman; N, normal; AB, abnormal; NA, not available; TS, transphenoidal surgery; HBsAg, hepatitis B surface antigen.

Based on the anatomical structure, we put forward that the underlying mechanism may be related to the communication between the hepatic portal vein system and the vena cava system, or the hematogenous pathway *via* lung to the brain. The cancer cell can metastasize to the pituitary through communication. In our case, we speculated that pituitary metastasis of HCC may be due to the location of the tumor and facilitate the metastasis. Furthermore, cancer cells may have an affinity to the pituitary. In those conditions, cancer cells are more likely to enter the circulation system and further metastasize to the pituitary. The molecular mechanism for pituitary metastasis of HCC needs to be further explored.

The appearance order of symptoms in the natural course may be useful for the confirmative diagnosis. On the one hand, the initial clinical symptoms in the clinical course may be related to the central nervous system (3, 6–10). Six cases have been reported in the literature. Those cases may present with a normal level of liver function and endocrine hormones. However, the symptoms of pituitary metastasis are not specific (13). Our case had a similar clinical course with neurological deficits as the initial symptoms. Therefore, the diagnosis may be difficult and may be misdiagnosed as a non-functioning pituitary adenoma. On the other hand, the symptoms of the HCC may be the initial clinical presentation. Metastasis of HCC to the pituitary gland may present with initial symptoms such as abdominal pain, ascites, and splenomegaly. When neurological symptom occurs, pituitary metastasis of HCC should be considered. Five cases have been reported in the literature (4, 11). In addition, gender predilection should also be taken into account. Almost all patients with pituitary metastasis of HCC are men. Diabetes insipidus is relatively more common in pituitary adenoma. When unspecific symptoms including fatigue, dizziness, and vomiting are present, pituitary metastasis should be taken into account (13).

The differential diagnosis of pituitary tumors needs the exclusion of diseases such as Rathke’s cleft cyst and aneurysm. The usual radiological imaging finding for pituitary metastasis of HCC with initial neurological symptoms is difficult to distinguish from pituitary adenoma (10, 14). Due to the higher cellular density and lower cytoplasm/nuclear ratio in metastases, the hypointensity in T2-weighted images may be helpful to distinguish metastases from chordoma-like tumors on MR. However, the partial pituitary tumor has similar characteristics (15). The feature of spectroscopy may provide useful information on the proliferation and hemorrhage of macroadenoma with dimensions over 20 mm. This indicates that positive findings of MR spectroscopy in the pituitary less than 20 mm suggest metastases other than pituitary adenoma (16). In addition, bone destruction is common in pituitary metastases, like our case. Arterial spin-labeling (ASL) in MR may also be a useful tool to distinguish pituitary metastases from the pituitary. The hyper-perfusion nature of the tumor in metastases is more common than in the pituitary adenoma (10).

The final diagnosis relies mainly on hematoxylin and eosin and immunohistochemical analysis (7). For pituitary tumor with unspecific characteristics, we think an intraoperative rapid pathological test is recommended, which can confirm the pathology and guide the extent of resection. Histopathologic findings of HCC include plump cells, deeply stained nucleoli, abundant eosinophilic cytoplasm, and specific immune markers. In our case, immunohistochemical analysis showed the positivity of PCK, Hepar, and glypican-3. Finally, the diagnosis of pituitary metastasis of HCC was confirmed.

There are no significant differences between surgical and non-surgical treatments for pituitary metastasis in survival time (17–19). Moreover, total tumor resection for pituitary metastasis is difficult to achieve due to its firm, diffuse, invasive, vascular, and hemorrhagic characteristics (20, 21). However, surgical decompression, such as ethanol injection and transcatheter arterial embolization, can alleviate symptoms and improve life quality (20). The mean survival time of patients with pituitary metastasis is 5 months (22), and the main causes of death are linked to the primary disease (23). Consequently, the minimally invasive approach may be a reasonable option, especially for patients with confirmed pituitary metastasis of HCC. The transsphenoidal approach has been reported not to affect the survival rate (17). Tumor resection can not only achieve relief of symptoms such as deterioration in vision and eyeball movement but also provide pathologic confirmation for subsequent treatment plans (24). Endocrine disorders after surgery can be administered with hormone replacement therapy. Subsequent adjuvant therapies including chemotherapy, radiotherapy, and immunotherapy should be managed according to the patient’s condition.

In summary, we report a case of hepatocellular carcinoma metastasis to the pituitary gland as initial symptom. The appearance order of symptoms may be helpful to differentiate diagnosis. Pituitary metastasis should be considered in HCC patients when the tumor is located in the sellar region and the endocrinological assessment is normal.

## Data availability statement

The original contributions presented in the study are included in the article/**Supplementary Material**. Further inquiries can be directed to the corresponding author.

## Ethics statement

The studies involving human participants were reviewed and approved by Ethics Committee on Biomedical Research, West China Hospital of Sichuan University. The patients/participants provided their written informed consent to participate in this study. The animal study was reviewed and approved by Ethics Committee on Biomedical Research, West China Hospital of Sichuan University. Written informed consent was obtained from the participant/patient(s) for the publication of this case report.

## Author contributions

Conceptualization: SZ, BC. Data curation: FD, QH. Methodology: FD. Resources: BC, SZ. Supervision: SZ, CY. Validation: SZ. Writing—original draft: QH. Writing—review and editing: QH. All authors contributed to the article and approved the submitted version.

## References

[1] Fukutomi M Yokota M Chuman H Harada H Zaitsu Y Funakoshi A . Increased incidence of bone metastases in hepatocellular carcinoma. Eur J Gastroenterol Hepatol (2001) 13(9):1083–8. doi: 10.1097/00042737-200109000-00015 11564960

[2] He W Chen F Dalm B Kirby PA Greenlee JD . Metastatic involvement of the pituitary gland: a systematic review with pooled individual patient data analysis. Pituitary (2015) 18(1):159–68. doi: 10.1007/s11102-014-0552-2 24445565

[3] Aung TH Po YC Wong WK . Hepatocellular carcinoma with metastasis to the skull base, pituitary gland, sphenoid sinus, and cavernous sinus. Hong Kong Med J (2002) 8(1):48–51. 11861994

[4] Karamouzis MV Melachrinou M Fratzoglou M Labropoulou-Karatza C Kalofonos HP . Hepatocellular carcinoma metastasis in the pituitary gland: case report and review of the literature. J Neurooncol (2003) 63(2):173–7. doi: 10.1023/a:1023994604919 12825821

[5] Komninos J Vlassopoulou V Protopapa D Korfias S Kontogeorgos G Sakas DE . Tumors metastatic to the pituitary gland: case report and literature review. J Clin Endocrinol Metab (2004) 89(2):574–80. doi: 10.1210/jc.2003-030395 14764764

[6] Hirsch D Benbassat CA Drozd T Okon E Blum I . Pituitary and bilateral adrenal enlargement: an unusual presentation of hepatocellular carcinoma. J Endocrinol Invest (2005) 28(5):454–8. doi: 10.1007/BF03347227 16075930

[7] Moreno-Perez O Peiro FM Lopez P Boix E Meoro A Serna-Candel C . An isolated pituitary metastasis as presentation of a differentiated hepatocellular carcinoma mimicking a nonfunctioning macroadenoma. J Endocrinol Invest (2007) 30(5):428–33. doi: 10.1007/BF03346322 17598977

[8] Takigawa T Matsumaru Y Hayakawa M Ikeda K Matsumura A . Transarterial embolization with use of lipiodol and gelatin sponge for active nasal bleeding from hepatocellular carcinoma metastasis in the pituitary gland. Neurol Med Chir (Tokyo) (2011) 51(8):592–5. doi: 10.2176/nmc.51.592 21869584

[9] Wilson TC . Kirby PA. a 50-year-old man with back pain and a sellar mass. metastatic hepatocellular carcinoma. Brain Pathol (2013) 23(3):365–6. doi: 10.1111/bpa.12053 PMC8029350 23587145

[10] Tanaka T Hiramatsu K Nosaka T Saito Y Naito T Takahashi K . Pituitary metastasis of hepatocellular carcinoma presenting with panhypopituitarism: a case report. BMC Cancer (2015) 15:863. doi: 10.1186/s12885-015-1831-7 26545979 PMC4636744

[11] LAR G Mattogno PP Pompucci A Coli A Rigante M Mangiola A . An extremely rare case of a single isolated pituitary metastasis from hepatocellular carcinoma. J Neurosurg Sci (2017) 61(2):213–5. doi: 10.23736/S0390-5616.16.03252-5 26868267

[12] Ambalavanan J Peravali M Perry DJ . Rare case of hepatocellular carcinoma metastasising to the pituitary and cavernous sinus causing panhypopituitarism and bilateral ophthalmoplegia. BMJ Case Rep (2020) 13(10):e236377. doi: 10.1136/bcr-2020-236377 PMC7592250 33109695

[13] Peppa M Papaxoinis G Xiros N Raptis SA Economopoulos T Hadjidakis D . Panhypopituitarism due to metastases to the hypothalamus and the pituitary resulting from primary breast cancer: a case report and review of the literature. Clin Breast Cancer (2009) 9(4):E4–7. doi: 10.3816/CBC.2009.n.047 19933072

[14] Fassett DR Couldwell WT . Metastases to the pituitary gland. Neurosurg Focus (2004) 16(4):E8. doi: 10.3171/foc.2004.16.4.9 15191337

[15] Wong ET Lu XQ Devulapalli J Mahadevan A . Cyberknife radiosurgery for basal skull plasmacytoma. J Neuroimaging (2006) 16(4):361–3. doi: 10.1111/j.1552-6569.2006.00062.x 17032388

[16] Stadlbauer A Buchfelder M Nimsky C Saeger W Salomonowitz E Pinker K . Proton magnetic resonance spectroscopy in pituitary macroadenomas: preliminary results. J Neurosurg (2008) 109(2):306–12. doi: 10.3171/JNS/2008/109/8/0306 18671644

[17] Morita A Meyer FB Laws ER Jr . Symptomatic pituitary metastases. J Neurosurg (1998) 89(1):69–73. doi: 10.3171/jns.1998.89.1.0069 9647174

[18] Deng S Ruan D He J . Rare submandibular gland metastasis of hepatocellular carcinoma: case report and review of the literature. J Int Med Res (2021) 49(3):300060521997592. doi: 10.1177/0300060521997592 33719634 PMC7952849

[19] Tunc B Filik L Tezer-Filik I Sahin B . Brain metastasis of hepatocellular carcinoma: a case report and review of the literature. World J Gastroenterol (2004) 10(11):1688–9. doi: 10.3748/wjg.v10.i11.1688 PMC4572783 15162554

[20] Ruelle A Palladino M Andrioli GC . Pituitary metastases as presenting lesions of malignancy. J Neurosurg Sci (1992) 36(1):51–4. 1323647

[21] Sioutos P Yen V Arbit E . Pituitary gland metastases. Ann Surg Oncol (1996) 3(1):94–9. doi: 10.1007/BF02409058 8770309

[22] Kim SU Kim DY Park JY Ahn SH Nah HJ Chon CY . Hepatocellular carcinoma presenting with bone metastasis: clinical characteristics and prognostic factors. J Cancer Res Clin Oncol (2008) 134(12):1377–84. doi: 10.1007/s00432-008-0410-6 PMC12161720 18483745

[23] Hsieh CT Sun JM Tsai WC Tsai TH Chiang YH Liu MY . Skull metastasis from hepatocellular carcinoma. Acta Neurochir (Wien) (2007) 149(2):185–90. doi: 10.1007/s00701-006-1071-3 17180305

[24] Post KD . Pituitary metastases: what is the role of surgery? World Neurosurg (2013) 79(2):251–2. doi: 10.1016/j.wneu.2012.05.006 22634465

