# Supplementary figures and images for: Pituitary metastasis of hepatocellular carcinoma as the initial presentations: a case report and review of the literature
Source: Front Oncol. 2023 Jul 6;13:1123855. doi: 10.3389/fonc.2023.1123855 (PMC10358273; doi:10.3389/fonc.2023.1123855)

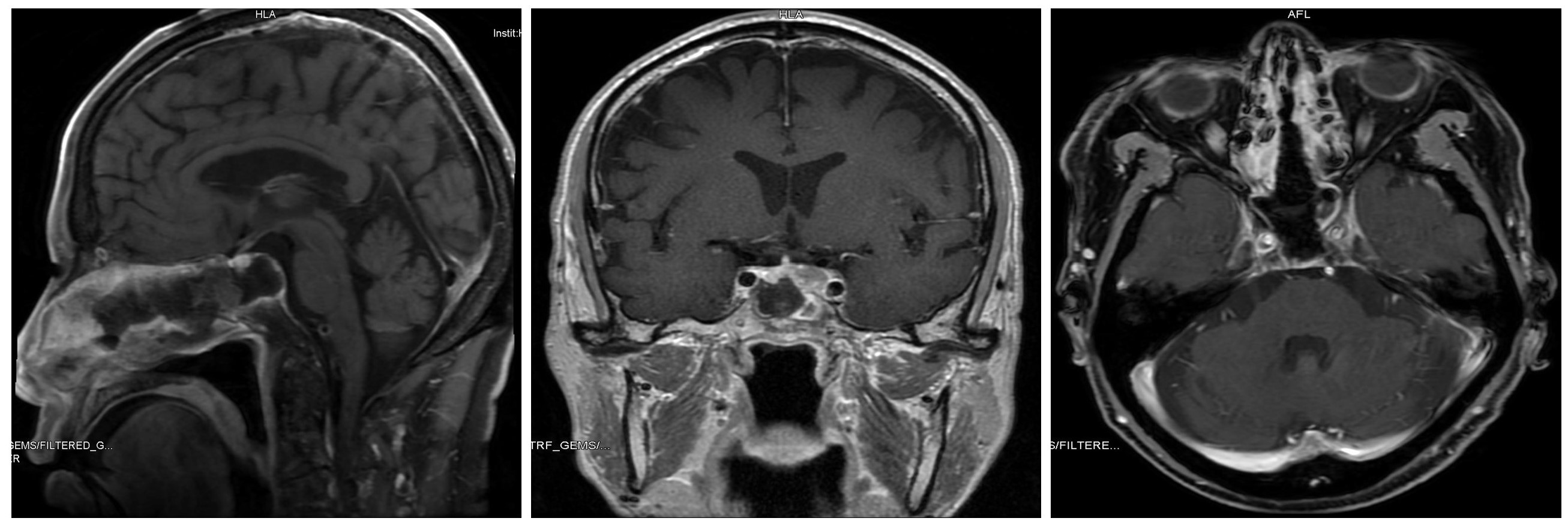

Supplement: Supplementary file 1 [file Image_1.jpeg]
